# Does COVID-19 cause an increased risk of hospitalization or death in patients with inflammatory rheumatic diseases treated with biological DMARDs or targeted synthetic DMARDs?

**DOI:** 10.1093/rap/rkaa061

**Published:** 2020-10-21

**Authors:** May Lwin, Christopher Holroyd, Dinny Wallis, Brian Davidson, Lyndsey Goulston, Hans de Graaf, Christopher J Edwards

**Affiliations:** NIHR Clinical Research Facility; Department of Rheumatology, University Hospital Southampton, Southampton, UK; Department of Rheumatology, University Hospital Southampton, Southampton, UK; Department of Rheumatology, University Hospital Southampton, Southampton, UK; NIHR Clinical Research Facility; NIHR Clinical Research Facility; NIHR Clinical Research Facility

**Key message**

•Poor outcomes from severe acute respiratory syndrome coronavirus-2 are not common for patients with inflammatory rheumatic diseases taking advanced therapies.

Dear  Editor, The world has been gripped by the severe acute respiratory syndrome coronavirus-2 (SARS-CoV-2) that causes coronavirus disease-2019 (COVID-19) and has been responsible for >38 000 deaths in the UK [1]. There are serious concerns that patients with inflammatory rheumatic diseases treated with advanced therapies, such as biological DMARDs (bDMARDs) and targeted synthetic DMARDs (tsDMARDs), might be at increased risk of the severe consequences of SARS-CoV-2. As a result, UK patients have been advised to shield or self-isolate to reduce the likelihood of infection [2].

To assess the degree of risk in this patient group, we used several methods to record cases of severe SARS-CoV-2 in our hospital. This included information from our advanced therapy database and clinics, the department telephone advice line and a real-time study of patient status and symptoms (Adult ImmunoCOVID study) [3]. The ImmunoCOVID study has recruited patients from the advanced therapy database recording possible COVID-19 symptoms (fever, cough, shortness of breath, blocked nose, red eye, fatigue, joint pain, muscle pain, nausea, diarrhoea and vomiting) and confirmed positive cases of COVID-19 on a weekly basis using an online portal. Using multiple sources of information gave us the greatest chance of capturing cases of infection leading to severe disease, including hospitalization and death. Ethics approval and informed consent were supplied via the ImmunoCOVID study (Research Ethics Committee (REC) ref. 20/YH/0110) and SMaRT biological therapy database (REC ref. 15/WS/0276).

During the period of 2 March 2020 to 22 May 2020, a total of 1004 patients with RA (*n* = 574), PsA (*n* = 204), axial spondyloarthropathy (*n* = 215) and SLE (*n* = 11) receiving advanced therapies were included in the advanced therapy database. There have been no deaths, but we have identified seven suspected COVID-19 cases that were swabbed and tested using a laboratory PCR-based system to determine the presence of active infection (three adalimumab, one abatacept, one certolizumab, one sarilumab and one ustekinumab). Only one of these patients had loss of sense of smell and taste. Two patients subsequently tested positive for SARS-CoV-2. An 81-year-old female with RA on abatacept and MTX, with co-morbidities including hypertension, ischaemic heart disease, atrial fibrillation and a previous pulmonary embolism, was admitted with fever and shortness of breath along with a cough and mild haemoptysis. Oxygen saturation was normal on air, and no antibiotics were required. She recovered and was discharged after 9 days. The second patient was a 52-year-old female with PsA on ustekinumab and MTX with no relevant co-morbidities, who presented with fever, cough, shortness of breath, chest tightness, ageusia, anosmia, diarrhoea and vomiting. Consolidation was present on chest X-ray, and she was admitted to hospital and treated with IV antibiotics (levofloxacin). No oxygen was required, and she was discharged after 3 days. In both patients, MTX and the bDMARD were suspended temporarily but have subsequently been restarted. Among the remaining five patients who tested negative, one patient had hypertension and was on ramipril 5 mg/day, and the other four patients had no co-morbidities. Given the multiple methods of recording cases, it seems unlikely that we will have missed serious cases of disease that resulted in admission to hospital or death. It is very possible that lack of widespread testing means that other patients will have had SARS-CoV-2 infection without serious disease. To put our numbers of patients affected in context, until 22 April 2020 a total of 455 patients had been admitted to University Hospital Southampton with COVID-19.

It appears that individuals with inflammatory rheumatic diseases receiving advanced therapies are not at significantly increased risk of hospitalization or death as a consequence of SARS-CoV-2 infection during a time of high viral prevalence in our hospital and the local community. The number of serious infections we have seen in this patient group is in line with the experience of colleagues working in Northern Italy [4] and, anecdotally, from other colleagues across the UK. The possibility remains that the low level of serious infection is attributable to the fact that the advice to shield and self-isolate has been very effective at reducing exposure. The chance that certain therapies used by patients with inflammatory rheumatic diseases, such as HCQ, tocilizumab and baricitinib, might be protective remains speculative until robust randomized controlled trials are concluded. 

*Funding:* No specific funding was received from any funding bodies in the public, commercial or not-for-profit sectors to carry out the work described in this manuscript.

*Disclosure statement*: C.J.E. has received honoraria, attended advisory boards, speakers bureau, and received research support from Abbvie, BMS, Biogen, Celgene, Fresenius, Gilead, GSK, Janssen, Lilly, Mundipharma, Pfizer, MSD, Novartis, Roche, Samsung, Sanofi, UCB. C.H. has received honoraria from Abbvie, Janssen, Lilly, Pfizer, MSD, Novartis, Roche, UCB. The other authors have declared no conflicts of interest.
